# A study to assess the unmet medical needs associated with the use of basal insulin in patients with type 2 diabetes

**DOI:** 10.1002/edm2.164

**Published:** 2020-10-31

**Authors:** Gustavo Frechtel, Lujan Forti, Cristina Faingold, Federico Perez Mangui, Silvia Orio, Claudia Issa, María S. Guaita, Norma Vivas, Julian A. De Luca

**Affiliations:** ^1^ Division Nutrition and Diabetes Sirio Libanés Hospital Buenos Aires Argentina; ^2^ SANOFI Buenos Aires Argentina; ^3^ Division of Endocrinology Cesar Milstein Hospital Buenos Aires Argentina; ^4^ CINME Centro de Investigaciones Metabólicas Buenos Aires Argentina; ^5^ IMOBA Investigaciones Médicas Buenos Aires Argentina; ^6^ Sanatorio Guemes Buenos Aires Argentina

**Keywords:** basal insulin, HbA1c, hypoglycaemia, type 2 diabetes

## Abstract

**Aim:**

To describe in a real‐world setting, the proportion of patients with a symptomatic hypoglycaemic event and the proportion of individuals with type 2 diabetes, who newly or recently initiated with basal insulin, achieving individual or general HbA1c target.

**Materials and Method:**

DINAS‐AR was a national prospective observational study to assess the unmet needs in patients with type 2 diabetes treated with basal insulin with or without oral antihyperglycaemic drugs and/or GLP‐1 receptor agonist. The study was conducted at 19 hospitals.

**Results:**

A total of 385 uncontrolled patients (≥18 years) who recently initiated basal insulin or who initiated treatment within a year prior to study enrolment entered the study. Outcomes were follow‐up incidence of hypoglycaemic events, change of HbA1C and achievement of HBA1c <7% or individual target. A total of 44 patients (11.9%) reported the occurrence of ≥1 symptomatic hypoglycaemia event(s). HbA1c reductions were greater in patients who had recently initiated treatment with basal insulin (between 15 and 90 days prior to study entry) vs patients who initiated treatment within 1 year. A total of 80 patients (31.6%) achieved individual HbA1c target (or target <7.0%) at Week 24. Furthermore, the proportion of patients achieving this target without symptomatic hypoglycaemia was 26.1% (n = 66). A lower percentage of glycemia target achievement was observed in patients reporting hypoglycaemia (n = 14), 20.6% of all patients reporting hypoglycaemia event(s) vs (n = 66) 35.7% of all patients without hypoglycaemia event reported.

**Conclusion:**

In this real‐world study, although the hypoglycaemia rate was not high in adults with type 2 diabetes treated with insulin, there was a lower percentage of patients that achieved glycemic target among those reporting hypoglycaemia events vs patients who did not report them.

## Graphical abstract

This real‐world study aims to describe the proportion of patients with a symptomatic hypoglycaemic event and the proportion of individuals with type 2 diabetes, who newly or recently initiated with basal insulin, achieving individual o general HbA1c target in Argentina. Although the hypoglycaemia rate was not high in adults with type 2 diabetes treated with insulin, there was a lower percentage of patients that achieved glycaemic target among those reporting hypoglycaemia events versus patients who did not report them.

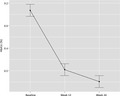

## 1 INTRODUCTION

Type 2 diabetes is a chronic condition, and its frequency is increasing mainly due to the impact of obesity growth.^1^ In Argentina, the diabetes prevalence reported in the last national risks factors survey, carried out every 4 years and evidenced an increase of around 30% in the last 10 years, showing a prevalence of 12.7% in 2018.^2^ A new international study using Markov model shows that by 2060, the number of US adults with diagnosed diabetes is projected to nearly triple, and the per cent prevalence rate will double.^3^ The burden of the disease is significant both to individuals and to the society as a whole. Its related costs are primarily triggered by late complications.^4^

Type 2 diabetes disease is characterized by progressive β‐cell dysfunction, and it requires the stepwise addition of several therapeutic strategies in order to achieve an adequate metabolic control. These interventions typically start with changes in lifestyle implemented right upon diagnosis and are followed by the initiation of oral antihyperglycaemic drugs and subsequently injectable therapies, including complete replacement of severely reduced endogenous insulin secretion.^5^

Evidence from previous interventional studies in diabetes has clearly shown that an adequate long‐term glycemic control plays an important role in reducing the risk of developing late complications.^6^ This is well established in the case of microvascular complications. The relationship between poor glycemic control and macrovascular complications is still a controversial issue to be largely discussed, although epidemiological studies have consistently shown that the poorer the glycemic control, the worse the cardiovascular outcomes.^7^ Therefore, the ultimate objective of the therapies administered is to attain adequate glycemic control aimed to delay or prevent late complications.^5^

In spite of the overwhelming evidence showing the crucial role of adequate glycemic control in the management of type 2 diabetes and despite the increasing number of antidiabetic drugs available for prescription, a considerable percentage of patients are still unable to reach the 7.0% HbA1c general target, which places them at a higher risk of complications.^8^ One of the reasons why achieving the goal is limited in the diabetic population is the potential impact of hypoglycaemia.

As the improvement in metabolic control is typically associated with a higher frequency of hypoglycaemia, both physician and patient are reluctant to achieve an adequate glycemic control. Physicians are not willing to expose their patients to an increased risk of hypoglycaemia while patients would also like to avoid the feared experience of an hypoglycaemic episode.

Different therapeutic agents are associated with different risk levels of hypoglycaemia.^5^ Undoubtedly, the antihyperglycaemic therapeutic approach with the highest risk of hypoglycaemia is insulin therapy. Therefore, it is not surprising that reluctance is high to initiate therapy and to optimize the therapeutic regimen used in the case of insulin therapy.^9^

Even in the simplest approach, in the case of basal insulin therapy, which is the most popular scheme to initiate insulin treatment for type 2 diabetes, the high level of clinical inertia results in late insulin treatment initiation and suboptimal dosing, both aspects being clearly associated with suboptimal general glycemic control in many type 2 diabetes patients.^8^, ^9^

Although there seems to be clinical consensus on the fact that treatment‐associated hypoglycaemia is a key factor leading to insulin late initiation and reluctance to optimize the dose (titration period) administered to reach the HbA1c target, evidence available to prove the relationship between treatment‐associated hypoglycaemia and failure to reach the glycemic target is surprisingly scarce.^10^

As the frequency of severe hypoglycaemia is relatively low in patients with type 2 diabetes on insulin therapy, it is extremely challenging to establish the relationship between severe hypoglycaemic events and the failure to reach the HbA1c target. On the other hand, nonsevere events, which might not be captured in some databases, are more common and can be terrifying and undesirable in real life, and therefore, such events could have a negative impact on target achievement.^11^

So, the question is, then, how to reliably capture nonsevere events in order to confirm those relationships in real clinical practice.

A possible way to record these nonsevere events is to monitor patients in clinical practice and ask them to record any hypoglycaemic event they undergo in real time.

The main purpose of this study is to reliably detect hypoglycaemic events in uncontrolled type 2 diabetes patients who recently initiated treatment with basal insulin or who initiated treatment within a year prior to study enrolment, and to establish the relationship between hypoglycaemic episodes occurring during the 24‐week observational period and the achievement of glycemic target. In addition, the study focused on describing the proportion of patients reaching their individual HbA1c target and/or general 7.0% glycemic target.

Establishing the link between nonsevere hypoglycaemic events and the failure to reach the glycemic target is important not only to confirm the assumption that both outcomes are related (hypoglycaemia is considered a safety outcome while reaching the HbA1c target is a surrogate outcome for late diabetes complications) but also to emphasize the importance of nonsevere hypoglycaemic events to health decision makers.

Nowadays, payers attribute cost implications only to severe hypoglycaemic events and associated nonsevere events with no direct healthcare costs. A confirmation that nonsevere hypoglycaemic events have a harmful effect on glycemic control could change this point of view and could help prescribing physicians to substantiate the need for new and better antihyperglycaemic drugs associated with a lower risk of hypoglycaemia, as those drugs, including new basal insulin preparations, are typically associated with the clinical benefit of reducing the frequency/rate of nonsevere hypoglycaemic events.

As hypoglycaemia could be associated with other undesired clinical and health economics outcomes,^12^ the study includes several secondary objectives to describe the potential impact of hypoglycaemia, with special focus on reliably collected nonsevere hypoglycaemic events, on factors such as weight gain, fear of hypoglycaemia, treatment adherence and discontinuation, and use of healthcare resources.

## 2 MATERIALS AND METHODS

### 2.1 Study design and participants

This study was a multicenter, prospective *follow‐up at week 24*, single‐cohort and noninterventional study, conducted at 19 hospital sites in Argentina. After informed consent signature, clinical and laboratory data (baseline) were captured for eligible patients and clinical visits were conducted according to local practice. Data were collected at study entry and at Weeks 12 and 24.

Written informed consent was obtained from each patient participating in the study by means an informed consent form (ICF) approved by each site EC/IRB before starting the study. All relevant aspects of the study were explained to the patient before obtaining informed consent and prior to carrying out any activity that is not part of routine care.

The study aimed to enrol 400 participants with T2DM ≥18 years of age who had recently initiated treatment with BI, human or analogue, at least 15 days before enrolment or were on treatment with BI for <12 months with or without oral antihyperglycaemic drugs and/or GLP‐1 receptor agonist.

Participants were required to have HbA1c between 7.5% and 11.0% (≥58 to ≤97 mmol/mol) for newly initiated BI users and between 7.5% and 10.0% (≥58 to ≤86 mmol/mol) for previously initiated BI users, and to be willing to perform self‐monitoring of blood glucose (SMBG) and to complete a patient diary. Exclusion criteria included treatment with rapid‐acting o premix insulin within the next 3 months. Participants were also excluded if they were more likely to have type 1 diabetes (<40 years old and had initiated insulin within 1 year of diabetes diagnosis), or if they were, or planning to become, pregnant.

To help eliminate bias, investigators were advised to include consecutive patients suitable for the study. Signed informed consent was obtained from all participants. To mirror real‐world clinical practice for the management of diabetes, no fixed study visit was scheduled during the follow‐up period, rather, clinical visits, including the possibility of phone visits, and treatment choices were undertaken according to local practice. At study entry, data were collected from participants concerning demographics, medical history, especially concerning diabetes complications, comorbidities and history of severe hypoglycaemia, and type of BI being used (human intermediate‐acting or long‐acting analogue). The use and titration of concomitant antidiabetic medications other than BI during the study period were left in the hands of the treating doctors. This study was observational, with treatment carried out according to local practice and conducted in accordance with the Declaration of Helsinki and the International Conference on Harmonization guidelines for Good Clinical Practice.

### 2.2 HbA1C targets

At baseline, an individualized long‐term HbA1c target was set for each participant by their physician. In the case of those for whom an individualized target was not set, a general HbA1c target less than 7.0% [<53 mmol/mol] was defined, based on current guidelines (Table 1). A separate 12‐week objective was set by physicians, based on the HbA1c level they anticipated patients would be able to reach by Week 12; however, the results of this objective are not the focus of this report.

**Table 1:** (a) Demographics and other baseline characteristics. (b) Drug therapy for diabetes (baseline)

Characteristic	Newly initiated (N = 236)	Previously initiated (N = 149)	Overall (N = 385)	*P*‐value
(a)				
Age (years), mean (SD)	60 (12.0)	61 (10.0)	60 (11.0)	.319
Sex (female/male)	116/120	83/66	199/186	.297
Body weight (kg), mean (SD)	83.64 (16.76)	83.36 (17.51)	83.53 (17.03)	.736
Body mass index (BMI) (kg/m^2^), mean (SD)	31.03 (5.83)	31.28 (5.87)	31.13 (5.84)	.673
Comorbidities, n (%)				
Arterial hypertension	140 (59.8%)	106 (71.6%)	246 (64.4%)	.019
Dyslipidaemia	129 (55.4%)	87 (58.8%)	216 (56.7%)	.511
Coronary heart disease	27 (11.7%)	10 (6.8%)	37 (9.8%)	.114
Acute myocardial infarction	12 (5.1%)	7 (4.7%)	19 (5.0%)	.850
Myocardial revascularization procedure	14 (6.0%)	7 (4.7%)	21 (5.5%)	.590
Any diabetes complication, n (%)	88 (37.3%)	61 (40.9%)	149 (38.7%)	.473
Diabetic neuropathy, n (%)	52 (22.4%)	31 (20.8%)	83 (21.8%)	.710
Diabetic retinopathy, n (%)	35 (15.0%)	32 (21.5%)	67 (17.5%)	.105
Leading to blindness, n (%)	1 (2.9%)	1 (3.1%)	2 (3.0%)	.948
Renal function impairment, n (%)	27 (11.5%)	25 (16.9%)	52 (13.6%)	.137
Related to diabetes, n (%)	22 (81.5%)	23 (92.0%)	45 (86.5%)	.266
Related to other conditions, n (%)	3 (11.1%)	0 (0.0%)	3 (5.8%)	.086
Microalbuminuria, n (%)	11 (40.7%)	11 (44.0%)	22 (42.3%)	.812
Macroalbuminuria, n (%)	7 (25.9%)	6 (24.0%)	13 (25.0%)	.872
Advanced kidney disease, n (%)	4 (14.8%)	3 (12.0%)	7 (13.5%)	.766
End‐stage renal failure, n (%)	0 (0.0%)	1 (4.0%)	1 (1.9%)	.294

Characteristic	Newly initiated (N = 236)	Previously initiated (N = 149)	Overall (N = 385)
(b)			
Basal insulin therapy			
Interval between initiation of current basal insulin and study entry (in days), mean (SD)	38 (20)	226 (85)	111 (107)
Type of basal insulin at screening, n (%)			
Human intermediate‐acting basal insulin	65 (27.5%)	54 (36.2%)	119 (30.9%)
Long‐acting basal analogue	124 (52.5%)	65 (43.6%)	189 (49.1%)
Ultra‐long‐lasting basal analogue	47 (19.9%)	30 (20.1%)	77 (20.0%)
Recommended way of titration, n (%)			
Physician‐driven	206 (91.2%)	120 (83.9%)	326 (88.4%)
Patient‐driven	20 (8.8%)	23 (16.1%)	43 (11.7%)
Oral antidiabetic medications (concomitant)			
Any antidiabetic medication received, n (%)	212 (89.8%)	131 (87.9%)	343 (89.1%)
By medication class, n (%)			
DPP‐IV inhibitors	68 (28.8%)	42 (28.2%)	110 (28.6%)
Meglitinides	1 (0.4%)	0 (0.0%)	1 (0.3%)
GLP‐1 receptor agonists	3 (1.3%)	7 (4.7%)	10 (2.6%)
Metformin	199 (84.3%)	127 (85.2%)	326 (84.7%)
SGLT‐2 inhibitors	14 (5.9%)	12 (8.1%)	26 (6.8%)
Sulfonylureas	35 (14.8%)	12 (8.1%)	47 (12.2%)

Note:

Percentages are based on the number of patients assessed in each group.

Abbreviations: IQR, interquartile range; IU, international unit; SD, standard deviation.

### 2.3 Objectives and end‐points

The main purpose of this study is to reliably detect hypoglycaemic events in uncontrolled type 2 diabetes in patients who recently initiated treatment with basal insulin or who initiated treatment within a year prior to study enrolment, and to establish the relationship between hypoglycaemic episodes occurring during the 24‐week observational period and the achievement of glycemic target. In addition, the study could focus on describing the proportion of patients reaching their individual HbA1c target and/or general 7.0% glycemic target.

1. Primary end‐point was to describe the proportion of patients with symptomatic hypoglycaemic event(s) at Week 12, being the period of greatest change in basal insulin dose to reach glycemic target (titration period) with a higher risk of hypoglycaemia

Secondary objectives:

1. To describe the proportion of patients reaching the general HbA1c target <7.0% at Week 24.
2. To describe the proportion of patients reaching the “24‐week HbA1c” target (defined as the HbA1c level expected to be reached by the patient at Week 24, as judged by the physician).
3. To describe the change in HbA1c from baseline to Week 24.
4. To describe the proportion of patients reaching the HbA1c target (individual or general target <7.0% if the individual target was not defined) without symptomatic hypoglycaemia at Week 24.
5. To describe the proportion of patients reaching the “24‐week HbA1c” target (defined as the HbA1c level expected to be reached by the patient at Week 24, as judged by the physician) without symptomatic hypoglycaemia.
6. To identify the screening visit factors associated with treatment failure, defined as the failure to reach the individual or general target <7.0% if the individual target was not defined, at Week 24.
7. To describe the changes in fasting plasma glucose (FPG) from baseline to Week 24, assessed by self‐measured plasma glucose (SMPG).
8. To describe the incidence of any nocturnal severe hypoglycaemic event and at 24 hours.
9. To describe the incidence of any nocturnal nonsevere symptomatic hypoglycaemic event and at 24 hours.
10. To describe the incidence of nocturnal documented symptomatic hypoglycaemic events and over 24 hours at Week 24.
11. To describe the incidence of hypoglycaemic events which lead to hospitalization at Week 24.
12. Change in basal insulin dose at Week 24.
13. Assessment of fear of hypoglycaemia by the patient—by completing the Hypoglycaemia Fear Survey II (HFS‐II).^13^ (Supplementary appendix page 7 and 8).

### 2.4 Data analysis and statistics

The sample size was determinated with an estimation based on a 95% (two‐sided) confidence interval of the percentage of patients with symptomatic hypoglycaemia at Week 12. Assuming an expected 30%, based on conservative estimates from previous randomized clinical trials (20%‐45%),^14^, ^15^ for patients with symptomatic hypoglycaemia at Week 12, and a 20% of nonevaluable patients, enrolling 400 patients allowed to estimate this percentage with an accuracy of at least 5% and a power >80%. Data were summarized with descriptive statistics. For continuous variables, results were expressed as number of patients, mean/standard deviation, or median/interquartile range (depending on variable distribution) and range, whereas the number of patients, frequency and percentage were described for categorical variables. Continuous variables were compared between groups using the paired sample t test or Wilcoxon signed rank test (depending on variable distribution), whereas categorical variables were compared using the Pearson *χ* ^2^ or Fisher's exact test, as appropriate. All tests will be two‐sided, and *P*‐values less than 0.05 were considered as statistically significant. The 95% confidence intervals were provided if relevant.

Missing data or unknown responses were not counted for percentages. For hypoglycaemia, patients with a missing value confirmed during the study were not analysed, and therefore, they were not counted as “without hypoglycaemia” in all analyses.

It is not planned to test any statistical hypothesis in a confirmatory sense. All estimates were described in their entirety and evaluated descriptively. Confidence intervals and p‐values were interpreted in the perspective of the exploratory nature of the study.

Missing data were not imputed for this study and were handled by eliminating from analysis of respective parameter including the affected variable in all patients.

All extreme values (outliers) were excluded from analysis. These were related to data entry errors from investigators not properly corrected (eg, entry of an HbA1c value in a SMBG data field). However, the quantity of outliers was low (outliers concerning to laboratory results were identified in less than 5% of study sample).

A multivariable analysis was conducted to explore study entry (baseline) predictive factors associated with the occurrence of symptomatic hypoglycaemia up to Week 12 in the study population. Four multivariable logistic regression models were used to analyse the impact of covariates (such as HbA1c, body weight, insulin dose, whether the patient has been recently initiated, diabetes years of evolution, renal or hepatic failure, and concomitant antihyperglycaemic medication) in the presence of hypoglycaemia.

## 3 RESULTS

### 3.1 Study population and baseline characteristics

#### 3.1.1 Study population

The study, conducted from March 2017 to January 2019, evaluated 431 patients from Argentina in 19 centres, finally Including 236 patients newly initiated and 149 previously initiated participants. Mean treatment duration for previously initiated participants was approximately 7.5 months. Overall, 11.7% of participants that self‐titrated their insulin compared with 88.3% whose titration was determined by physicians. The proportion of participants self‐titrated their insulin was 8.8% vs 16.1%, corresponding to newly initiated vs previously initiated, respectively (Supplementary Appendix Table 2).

#### 3.1.2 Baseline characteristics

The analysis evidenced no major clinical differences between newly and previously initiated participants in condition of age, weight, BMI, presence of at least one microvascular complication, diabetes neuropathy, diabetes‐related functional impairment or estimated glomerular filtration rate (eGFR). Cardiovascular and metabolic diseases were the most common comorbidities reported at overall level and within both groups. Among these, arterial hypertension, dyslipaemia, fatty liver disease and coronary heart disease were the most frequent conditions reported. Besides, there was a higher proportion of hypertensive patients in the group of patients with previous initiation of basal insulin (*P* = .019) and no significant differences were observed between groups for the rest of comorbid conditions. Furthermore, no differences were observed in terms of the burden (coexistence) of comorbidities in a patient (*P* = .779). In this regard, the overall burden of comorbidities (excluding diabetes complications) showed a median of two comorbid conditions per patients in both groups. A total of 149 patients (38.7%) presented diagnosis of, at least, one complication from diabetes (ie, diabetic retinopathy, nephropathy and neuropathy) at the time of study entry. Among diabetes complications, diabetic neuropathy was the most common (n = 83, 21.8%) and no significant differences were observed between groups (Table 1a).

Most of the patients (n = 343, 89.1%) reported the concomitant use of, at least, one oral antidiabetic medication. Comparison among medication classes showed that metformin was the most common medication used (n = 326, 84.7%), at an overall level and in both groups, followed by DPP‐IV inhibitors (n = 110, 28.6%).

Comparative analysis by group revealed that sulfonylureas were received in higher proportion by patients who recently initiated basal insulin (*P* = .047), whereas no statistically significant differences between groups were found in the use of the rest of oral antidiabetic medications (Table 1b).

### 3.2 Primary end‐point

Analysis from study data revealed that a total of 44 patients (11.9%; 95% CI: 8.8% to 15.6%) reported the occurrence of, at least, one event of symptomatic hypoglycaemia at Week 12.

About half of these patients (n = 21, 47.7%) reported the occurrence of only one hypoglycaemic event, whereas 12 patients (27.3%) reported the occurrence of two hypoglycaemic events throughout the 12‐week follow‐up period.

Most of the patients that reported hypoglycaemic event(s) were those who recently initiated basal insulin (n = 27, 12,1%; 95% CI: 8.1%‐17.1%), whereas hypoglycaemic event(s) were reported in 17 patients with previous initiation of basal insulin (11.5%; 95% CI: 6.8%‐17.8%); however, no statistically significant difference between groups was found in the proportion of patients developing hypoglycaemic event(s) up to Week 12 (*P* = .856).

A multivariable analysis was conducted to explore study entry (baseline) predictive factors associated with the occurrence of symptomatic hypoglycaemia up to Week 12 in the study population. Multivariable analysis showed that increase in body weight (OR: 0.969; 95% CI: 0.947‐0.992; *P* = .008) reduces the odds, whereas the increase in basal insulin daily dose (OR: 1.024; 95% CI: 0.019‐1.051; *P* = .005) increases the odds of symptomatic hypoglycaemia (Supplementary Appendix; page 4 and 5).

### 3.3 Secondary end‐points

#### 3.3.1 Basal insulin therapy at Week 24

Overall, the average interval between the initiation of current basal insulin therapy and study entry was of 111 days (SD: 107).

In about half of patients (n = 189, 49.1%), a long‐acting basal analogue was the type of insulin prescribed, being the most common insulin type used in both groups.

Mean daily insulin dose (standard deviation [SD]) at baseline was 23 (15) U and 32 (17) U for newly and previously initiated patients, respectively. The majority of newly initiated (81.6%) and previously initiated (66.4%) participants were using once BI dosing. In most patients (n = 326, 88.4%), the titration method of basal insulin was driven by the physician and, in about half of patients (n = 164, 51.4%), a 2‐IU‐dose increment was recommended for each titration step. At Week 24, 2.3% in the newly initiated group and 1.4% in the previously initiated group discontinued insulin use during the study because of insufficient control (1.1%) and hypoglycaemia (0.8%). By Week 24, the daily insulin dose increased by an average of 4.9 U in both newly and previously initiated participants (Tables 2 and 3, Supplementary Appendix). There was a modest increase in weight over 24 weeks in the newly initiated participants +0.87 kg, and in the previously initiated patients, a small reduction of −0.16 kg was observed (Table 4, Supplementary Appendix).

**Table 2:** Change in mean and by group HbA1c

Group	HbA1c (in %)^a^ mean (SD)			Difference Week 24—baseline
	Baseline	Week 12	Week 24	
Overall	9.08 (1.25)	8.02 (1.36)	7.81 (1.43)	−1.27
Newly initiated	9.33 (1.13)	8.13 (1.59)	7.78 (1.43)	−1.55
Previously initiated	8.61 (1.26)	7.88 (0.98)	7.84 (1.43)	−0.77

^a^ Data analysed from patients presenting HbA1c test results in all study visits (n = 208)

**Table 3:** Change in mean FPG (measure by SMGB)

Group	SMBG (in mg/dL)^a^ mean (SD)			Difference Week 24—baseline
	Baseline	Week 12	Week 24	
Overall	163.53 (48.68)	149.84 (44.79)	145.05 (46.06)	−18.48
Recent initiation of basal insulin	169.12 (57.98)	155.91 (51.56)	149.90 (51.58)	−19.22
Previous initiation of basal insulin	156.32 (31.93)	142.02 (32.79)	138.80 (37.11)	−17.52

^a^ Data analysed for patients presenting SMBG results in all study visits (n = 222).

**Table 4:** Change in mean FPG (measure by laboratory)

Group	FPG (in mg/dL)^a^ mean (SD)			Difference Week 24—baseline
	Baseline	Week 12	Week 24	
Overall	176.18 (67.97)	154.35 (54.73)	144.97 (50.03)	−31.21
Recent initiation of basal insulin	192.94 (75.36)	160.74 (61.89)	146.07 (50.16)	−46.87
Previous initiation of basal insulin	154.91 (50.14)	146.24 (43.03)	143.57 (50.15)	−11.34

^a^ Data analysed for patients presenting FPG results in all study visits (n = 186).

#### 3.3.2 Change in HbA1c, fasting glucose (measure by SMPG) and FPG (measure by laboratory) at Week 24

**Figure 1:**
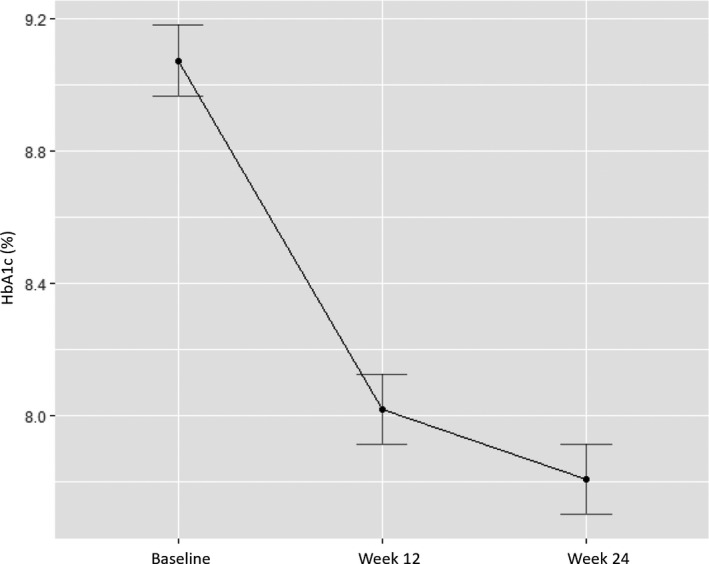
Change in mean HbA1c from baseline to Week 24

At Week 24, HbA1c had reduced from baseline by 1.55% in newly initiated participants and by 0.8% in previously initiated participants without significance from Week 12 to Week 24 (*P* = .065; Table 2, Figure 1).

A statistical significant drop (−18.48 mg/dL) in the mean FPG value (measure by SMPG) from baseline to Week 24 was observed. Post hoc analysis showed a significant drop in mean FPG from baseline to Week 12 (*P* < .001); however, there was no significance in the drop observed from Week 12 to Week 24 (*P* = .44; Table 3). A major fasting glucose decrease (−31.24 mg/dL) was observed when it was analysed in the laboratory at Week 24. Post hoc analysis showed that a statistical significant drop was observed in the mean value from baseline to Week 12 (*P* < .001); however, no significance was observed in the drop from Week 12 to Week 24 (*P* = .064; Table 4).

#### 3.3.3 Achievement of HbA1c targets at week 24

A total of 68 patients (26.9%) achieved both general HbA1c target <7.0% and HbA1c level defined for the patient, as judged by the physician. Among these patients, a slightly higher proportion in target achievement was observed in the group of patients who recently initiated basal insulin (Table 5). Furthermore, the proportion of patients achieving the target defined for the patient without symptomatic hypoglycaemia was of 22.9% (n = 58).

**Table 5:** Achievement of HbA1c targets at Week 24 (secondary end‐points)

Characteristic	Recent initiation of basal insulin (n = 147, %)	Previous initiation of basal insulin (n = 106, %)	Overall (N = 253, %)
Achieved HbA1c target (individual or general target <7.0% if the individual target was not defined), n (%)	51 (34.7)	29 (27.4)	80 (31.6)
Achieved general HbA1c target <7.0%, n (%)	42 (28.6)	26 (24.5)	68 (26.9)
Achieved HbA1c level (defined for the patient by physician), n (%)	44 (29.9)	24 (22.6)	68 (26.9)
Achieved HbA1c target (individual or general target <7.0% if the individual target was not defined) without symptomatic hypoglycaemia, n (%)	42 (28.6)	24 (22.6)	66 (26.1)
Achieved HbA1c level (defined for the patient by physician) without symptomatic hypoglycaemia, n (%)	37 (25.2)	21 (19.8)	58 (22.9)

A total of 80 patients (31.6%) achieved individual HbA1c target (or general target <7.0% if individual target was not defined) at Week 24. Among these, a slightly higher proportion was observed in the group of patients who had a recent initiation of basal insulin (Table 5). Furthermore, the proportion of patients achieving this target without symptomatic hypoglycaemia was of 26.1% (n = 66).

Lack of adherence to lifestyle recommendations (n = 80, 43.2% of patients assessed at Week 24) was reported as the main reason why HbA1c target was not achieved, followed by the lack of adherence to titration (n = 36, 19.5%)

Comparative analysis according to the occurrence of hypoglycaemic event(s) evidenced a lower proportion of glycemic target achievement in the group of patients who reported hypoglycaemia (Table 6). A lower percentage of glycemia target achievement was observed in patients reporting hypoglycaemia (n = 14), 20.6% of all patients reporting hypoglycaemia event(s) vs (n = 66) 35.7% of all patients without hypoglycaemia event reported (Table 7).

**Table 6:** Comparison of HbA1c target achievement at Week 24 per hypoglycaemia occurrence

Characteristic	With hypoglycaemia^a^ (n = 68, %)	Without hypoglycaemia (n = 185, %)
Achieved general HbA1c target <7.0%, n (%)	13 (19.1)	55 (29.7)
Achieved HbA1c level (defined for the patient by physician), n (%)	10 (14.7)	58 (31.4)
Achieved HbA1c level (individual or general target <7.0% if individual target was not defined), n (%)	14 (20.6)	66 (35.7)

^a^ Includes patients who reported, at least, 1 hypoglycaemia event from screening visit and up to Week 24.

**Table 7:** Comparative analysis of concomitant antidiabetic medications^a^

Medication class n (%)	Recent initiation of basal insulin (n = 236, %)	Previous initiation of basal insulin (n = 149, %)	Overall (N = 385, %, %)	*P*‐value
Any antidiabetic medication received	212 (89.8)	31 (87.9)	43 (89.1)	.558
By medication class				
DPP‐IV inhibitors	68 (28.8)	42 (28.2)	110 (28.6)	.894
Meglitinides	1 (0.4)	0 (0.0)	1 (0.3)	.426
GLP‐1 receptor agonists	3 (1.3)	7 (4.7)	10 (2.6)	.051
Metformin (Biguanides)	199 (84.3)	127 (85.2)	326 (84.7)	.808
SGLT‐2 inhibitors	14 (5.9)	12 (8.1)	26 (6.8)	.419
Sulfonylureas	35 (14.8)	12 (8.1)	47 (12.2)	.047
Thiazolidinediones	1 (0.4)	0 (0.0)	1 (0.3)	.426
Amylin analogues	0 (0.0)	0 (0.0)	0 (0.0)	n/a
Alpha‐glucosidase inhibitors	0 (0.0)	0 (0.0)	0 (0.0)	n/a

Note

Percentages are based on the total number of patients assessed in each group.

Abbreviation: n/a, not applicable.

^a^ Comprises medications received at any moment during the study period.

#### 3.3.4 Hypoglycaemic events

There were no severe hypoglycaemic events reported during the study.

A total of 68 patients reported the occurrence of, at least, one nonsevere symptomatic hypoglycaemic event (at any time of the day) up to Week 24, with an overall cumulative incidence of 0.176. About half of these patients (n = 33, 48.5%) reported the occurrence of only one hypoglycaemic event, whereas 14 patients (20.6%) reported the occurrence of two hypoglycaemic events during the 12‐week period. Furthermore, a total of five patients reported the occurrence of, at least, one nocturnal hypoglycaemic event up to Week 24, with an overall cumulative incidence of 0.012. No statistically significant difference was found between groups in the proportion of patients developing nonsevere hypoglycaemic event(s) up to Week 24 (*P* = .396), and no symptomatic hypoglycaemic events leading to hospitalization were reported during the study.

#### 3.3.5 Change in fear of hypoglycaemia

A nonsignificant decrease in average HFS‐II^13^ score from baseline to Week 24 has been observed. For the “Worry” scale, a −0.075 difference was observed (95% CI: −0.159 to 0.009; *P* = .082), whereas the decrease in the “Behaviour” scale was −0.023 (95% CI: −0.116 to 0.069; *P* = .617) (Table 8).

**Table 8:** Change in HFS‐II^13^

Characteristic	“Worry” scale		“Behaviour” scale	
	Baseline	Week 24	Baseline	Week 24
Mean (SD)	0.999 (0.932)	0.925 (0.833)	0.864 (0.808)	0.841 (0.819)
Median	1.00	1.00	0.800	0.800
Min‐max	0.00‐4.00	0.00‐4.00	0.00‐4.00	0.00‐4.00

## 4 DISCUSSION

The DINAS‐AR study was an observational, national, prospective real‐life study that assessed the achievement of HbA1c targets set by physicians based on individual patient characteristics. This study is a local adaptation of the DUNE study (The Diabetes Unmet Need with basal insulin Evaluation).^12^ Unlike DUNE, which is a multinational study in DINAS‐AR, only Argentine centres participated, and the primary objective was different. In the DUNE study, the objective was to describe the proportion of patients who achieved individualized or general HbA1c targets at Week 12, and in the DINAS‐AR study, the main objective was to describe the proportion of patients with a symptomatic hypoglycaemic event. But both studies evaluate similar secondary end‐points, and the inclusion criteria are very similar, so it results interesting to make the comparison between these two populations. In both registers, a greater reduction in HbA1c in newly and previously initiated patients was observed in the first 12 weeks, followed by a slight reduction between Week 12 and Week 24.

A total of 31.6% achieved individual HbA1c target (or general target <7.0% if individual target was not defined) at Week 24, similar to DUNE study and other real‐world studies.^16^, ^17^ This may be related, in part, to insufficient insulin dose titration during the total study period as well as during the titration period. In the Dune study, dose increases of 9 U and 5 U reported in the newly or previously initiated groups, respectively, were observed. Very similar results were found in DINAS‐AR, showing the daily basal insulin dose increased by an average of 4.9 U in both newly and previously initiated participants at Week 24, being this absence of intensive titration (titration inertia) reported previously in real‐world clinical practice.^17^

In randomized clinical trials such us the Bright study that compare the efficacy and safety of insulin glargine 300 units/mL (Gla‐300) vs insulin degludec 100 units/mL (IDeg‐100), the mean dose increases from baseline to Week 24 were 33.6 ± 24.4 units (0.36 ± 0.25 units/kg) and 29.1 ± 23.3 units (0.31 ± 0.24 units/kg) for Gla‐300 and IDeg‐100, respectively.^18^ Similar results can be observed in other RCTs such as EDITION 3, in which the efficacy and safety of Glargina u100 vs Glargina U300 is evaluated in naive patients.^14^ Unlike other real‐life studies, in most patients (n = 326, 88.4%), the titration method of basal insulin was driven by the physician.

Analysis from study data revealed that a total of 44 patients (11.9%; 95% CI: 8.8% to 15.6%) reported the occurrence of, at least, one event of symptomatic hypoglycaemia at Week 12. This results were lower than predicted (20%‐45%) based on conservative estimates from previous randomized clinical trials.^14^, ^15^ The limited increase in insulin dose in this study may have further contributed to the observed low incidence and rates of hypoglycaemia, as in the DUNE study.

The DINAS‐AR study may have been limited by several factors. Firstly, most of the medical investigators who treated these patients work in centres of excellence and are specialists; therefore, it could be assumed that the rate of patients in HbA1c target would be lower. Furthermore, hypoglycaemia data were collected by physicians based on patient diaries, which may be subject to recall bias.^19^

Additionally, the short observational period may also reduce the generalizability of the results, and the association between hypoglycaemia and target achievement may not necessarily have persisted over a longer observational period. Finally, the low rate of hypoglycaemia in the DINAS‐AR study has impact in the reported association between HbA1c target achievement and the occurrence, frequency and severity of hypoglycaemia. There is no information available on what device the patients used for their insulin administration (pen or vial/syringe).

Despite its limitations, the DINAS‐AR study provides relevant data in real‐life clinical practice in our country, particularly using the individualized HbA1c target defined by physicians.

## 5 CONCLUSIONS

In this study, we quantified hypoglycaemia events in a sample of patients with type 2 diabetes who recently initiated treatment with basal insulin or who initiated treatment within a year prior to study entry, who were followed up during a 24‐week period. Hypoglycaemic event(s) were reported in 11.9% (95% CI: 8.9%‐15.6%) of patients assessed at Week 12 and 18.7% of patients assessed at Week 24 while keeping similar proportions between both groups. Neither severe nor events requiring hospitalization were reported. Body weight reduction and basal insulin dose increase were identified as predictive factors for hypoglycaemia in a multivariable analysis. We also explored the relationship between the occurrence of hypoglycaemia and the achievement of glycemic (HbA1c) target at Week 24. In this regard, statistically significant drops in the HbA1c and FPG values were observed throughout the study follow‐up period. HbA1c target defined for the patient by physician (or general target <7.0% if not defined) was achieved by 31.6% (n = 80) of all patients assessed at Week 24; however, among these patients, a lower percentage of glycemia target achievement was observed in patients reporting hypoglycaemia (n = 14, 20.6% of all patients reporting hypoglycaemia event(s) vs n = 66, 35.7% of all patients without hypoglycaemia event reported) As a conclusion, it would be critical to avoid episodes of hypoglycaemia, not only because of the costs and complications that this brings, but also to ensure that more patients reach the glycemic goal.

## CONFLICT OF INTEREST

GF has served on advisory panels for Novo Nordisk, Sanofi and AstraZeneca. CF has served on advisory panels for Sanofi, Praxis Pharmaceutical, AstraZeneca, Novo Nordisk and MSD, and has received speaker fee from Eli Lilly, Sanofi, Novartis and Novo Nordisk. FPM has served on advisory panels for Sanofi, Boehringer Ingelheim and Eli Lilly. CI has no conflicts of interest to declare. LF, JAD and MSG are all employees of medical department of Sanofi.

## AUTHOR CONTRIBUTIONS

ANOVA SRL was involved in the statistical analysis of data. All authors were participating in the interpretation of data and writing, reviewing and ending of the manuscript, and all had final responsibility for approving the published version. ANOVA SRL is the guarantor of this work and, as such, had full access to all the data in the study and takes responsibility for the integrity of the data and the accuracy of the data analysis.

## Supporting information

Supplementary Appendix
