# Supplementary material for: A study to assess the unmet medical needs associated with the use of basal insulin in patients with type 2 diabetes
Source: Endocrinol Diabetes Metab. 2020 Oct 31;4(1):e00164. doi: 10.1002/edm2.164 (PMC7831225; doi:10.1002/edm2.164)
Supplement: Supplementary file 1 — Supplementary Appendix [file EDM2-4-e00164-s001.docx]

**SUPPLEMENTARY APPENDIX**

# INVESTIGATORS AND STUDY ADMINISTRATIVE STRUCTURE

This study was conducted in sites from Argentina and participating investigators included general practitioners (who have patients with type 2 diabetes and are acquainted with their insulin management) and diabetologists. Table 1 lists the principal investigators who participated in the study:

Table 1. List of investigators

| **Site ID** | **Country** | **Site name** | **Last name** | **First name** |
| --- | --- | --- | --- | --- |
| 001 | Argentina | IMOBA Investigaciones Médicas | Orio | Silvia |
| 002 | Argentina | Consultorio de Endocrinología, Diabetes y Metabolismo Dr. Faure y Col. | Castaño | Patricia |
| 003 | Argentina | Hospital Sirio Libanés | Fretchel | Gustavo |
| 004 | Argentina | Unidad Asistencial Dr. Cesar Milstein | Faingold | Cristina |
| 005 | Argentina | Maffei Centro Médico | Maffei | Laura |
| 006 | Argentina | Sanatorio Norte | Abalos | Franklin |
| 007 | Argentina | Sanatorio 9 de Julio | Carreño | Susana |
| 008 | Argentina | CIC WM | Müller | Santiago |
| 009 | Argentina | Hospital Privado de la Comunidad | Viñes | Gloria |
| 010 | Argentina | Sanatorio Güemes | Issa | Claudia |
| 011 | Argentina | CINME Centro de Investigaciones Metabólicas | Perez Manghi | Federico |
| 012 | Argentina | Consultorio Privado | Moisello | Alejandra |
| 013 | Argentina | Consultorio Privado | Lamela | Claudia |
| 014 | Argentina | Centro de Diabetes y Obesidad | Pons | Juan Carlos |
| 015 | Argentina | ICBA Instituto Cardiovascular de Buenos Aires | Sanabria | Hugo |
| 016 | Argentina | Sanatorio Parque | Saavedra | Silvia |
| 017 | Argentina | Centro Médico Viamonte | Aizenberg | Diego |
| 018 | Argentina | CEDIC | Gelersztein | Elizabeth |
| 019 | Argentina | Centro de Salud e Investigaciones Médicas | Mayer | Marcos |

**Table 2:** Basal insulin therapy history

| **Characteristic** | **Recent initiation of basal insulin (n=236)** | **Previous initiation of basal insulin (n=149)** | **Overall**  **(N=385)** |
| --- | --- | --- | --- |
| **Interval between initiation of current basal insulin and study entry (in days)** |  |  |  |
| mean (SD) | 38 (20) | 226 (85) | 111 (107) |
| median (IQR) | 32 (32) | 219 (159) | 62 (150) |
| min – max (range) | 15 – 88 (73) | 93 – 362 (269) | 15 – 362 (347) |
| **Type of basal insulin at screening, n (%)** |  |  |  |
| human intermediate-acting basal insulin | 65 (27.5%) | 54 (36.2%) | 119 (30.9%) |
| long-acting basal analogue | 124 (52.5%) | 65 (43.6%) | 189 (49.1%) |
| ultra-long-lasting basal analogue | 47 (19.9%) | 30 (20.1%) | 77 (20.0%) |
| **Recommended way of titration, n (%)** |  |  |  |
| physician-driven **^†^** | 206 (91.2%) | 120 (83.9%) | 326 (88.3%) |
| patient-driven **^‡^** | 20 (8.8%) | 23 (16.1%) | 43 (11.7%) |

*Note: Percentages are based on the number of patients assessed in each group; SD: standard deviation; IQR: interquartile range; IU: international unit*

*^†^ Titration is adapted by the physician at each contact – visit or phone – with the patient*

*^‡^ Patient has been instructed by the physician to self-titrate their basal insulin*

**Table 3:** Change in basal insulin dose

| **Group** | **Basal insulin dose (in IU)** ^†^  mean (SD) | | | **Difference Week 24 – Baseline** |
| --- | --- | --- | --- | --- |
|  | **Baseline** | **Week 12** | **Week 24** |  |
| **Overall** | 26.95 (17.32) | 30.54 (18.52) | 31.85 (19.11) | **+4.9** |
| **Recent initiation of basal insulin** | 23.40 (15.96) | 27.53 (17.52) | 28.82 (18.39) | **+5.42** |
| **Previous initiation of basal insulin** | 32.09 (17.98) | 34.91 (19.13) | 36.26 (19.35) | **+4.17** |

Table 4. Change in body weight

| **Group** | **Body weight (in kg)** ^†^  mean (SD) | | | **Difference Week 24 – Baseline** |
| --- | --- | --- | --- | --- |
|  | **Baseline** | **Week 12** | **Week 24** |  |
| **Overall** | 83.58 (17.29) | 83.76 (16.79) | 83.98 (16.68) | **+0.4** |
| **Recent initiation of basal insulin** | 83.61 (17.20) | 84.09 (16.76) | 84.48 (16.65) | **+0.87** |
| **Previous initiation of basal insulin** | 83.43 (17.47) | 83.30 (16.87) | 83.27 (16.75) | **-0.16** |

*† Data analyzed for patients presenting body weight results in all study visits (n=343)*

Figure 1. Overall study scheme


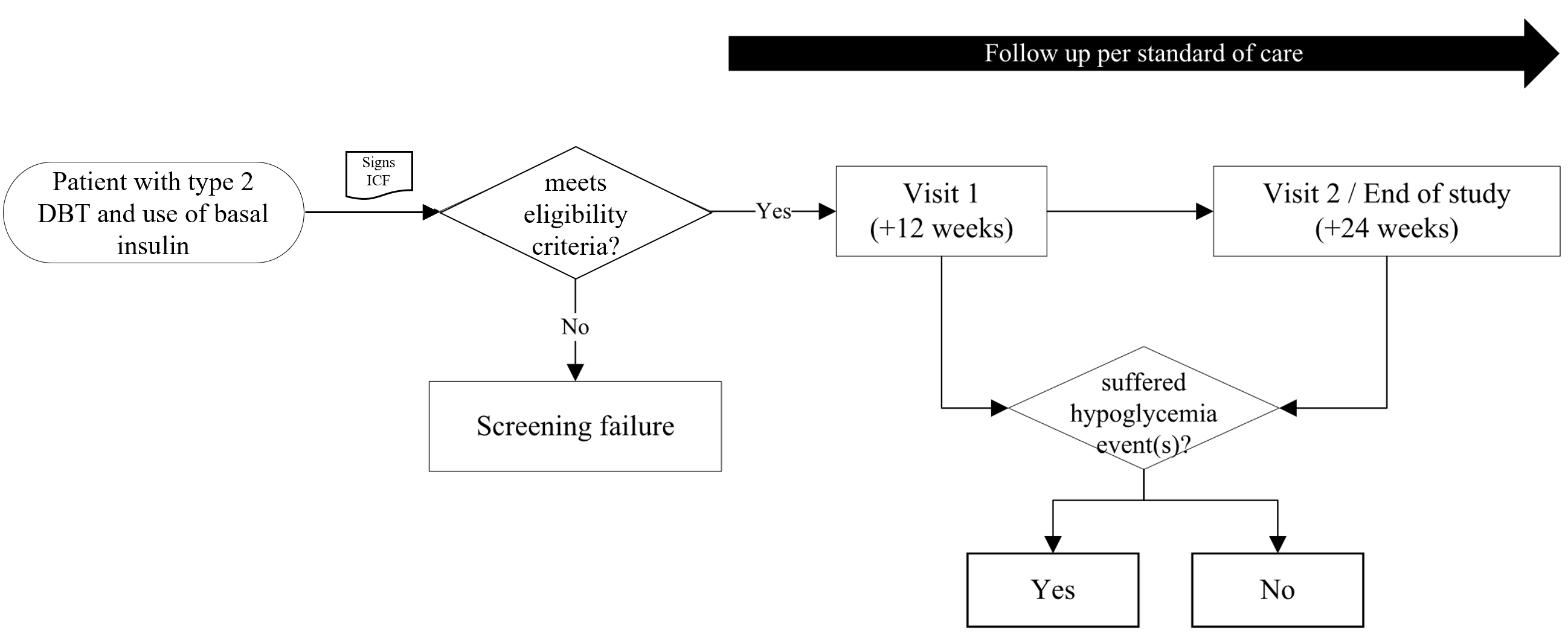


**Figure 2.** Disposition of patients (per group)


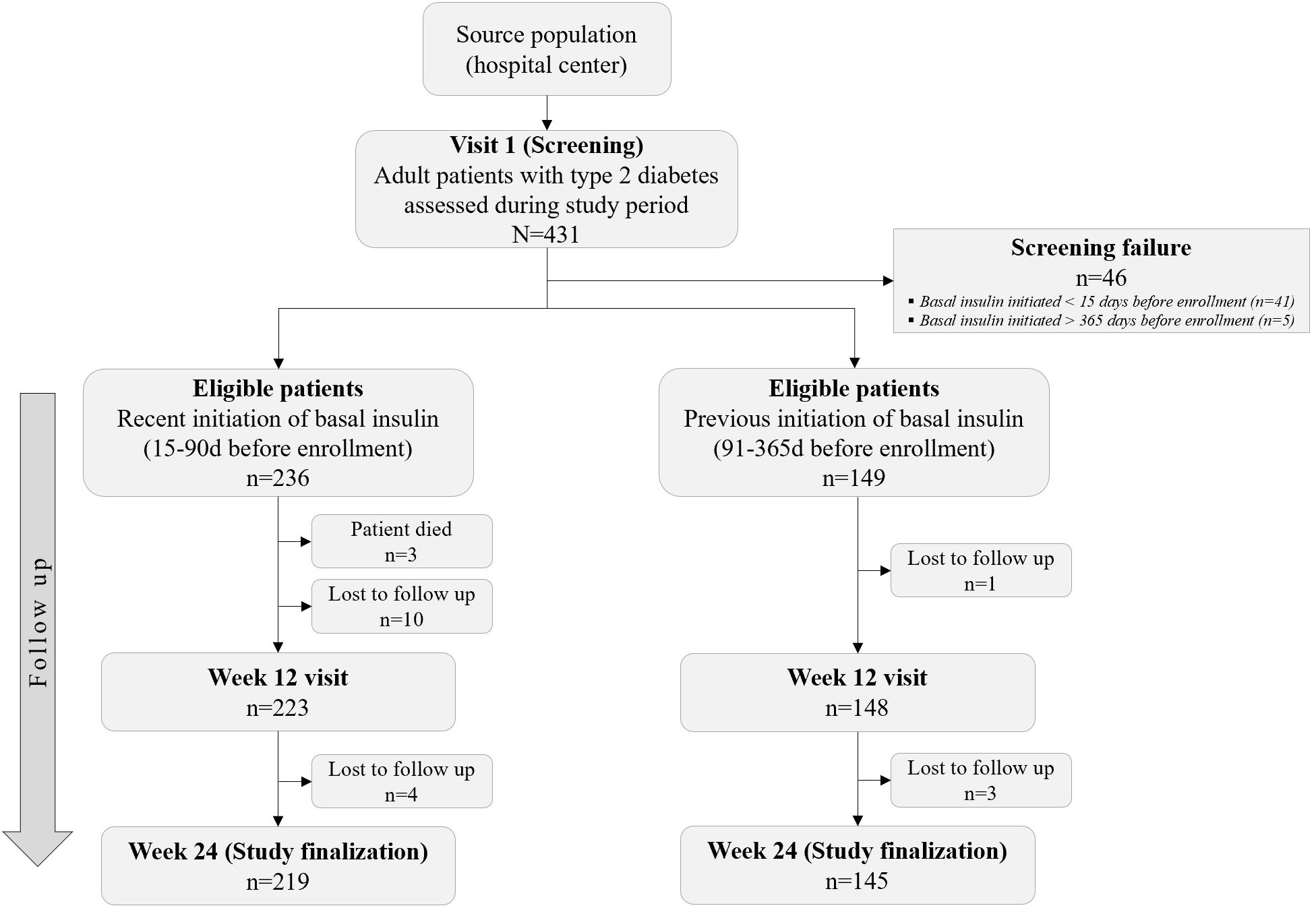


Figure 3. Difference in HbA1c between groups*.*

Previously initiated

Newly initiated


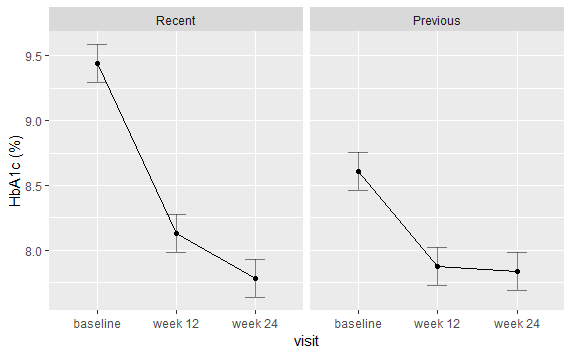


**METHODS - STATISTICAL ANALYSIS**

**Week 12.** A multivariable analysis was conducted to explore study entry (baseline) predictive factors associated with the occurrence of symptomatic hypoglycemia up to Week 12 in the study population. Analysis was made by logistic regression models to estimate odds ratios associated with the occurrence of symptomatic hypoglycemia. Clinically relevant variables, such as: HbA1c, body weight, basal insulin dose, time since diabetes diagnosis, renal compromise, fatty liver disease, use of concomitant antidiabetic medication(s) and time since initiation of basal insulin (group) were incorporated in the models. Modeling was assessed by goodness-of-fit (AIC, Hosmer-Lemeshow test), multicollinearity and confounding. The fitted model assessed was:

logit {P (X_i_)} = *α* + *β*^HBPR_0^_i_ + *β*^PESO^_i_ + *β*^YEVDBT^_i_ + *β*^IBDOS_0^_i_ + *β*^RENDBT_0^_i_ + *β*^ESTEAT_0^_i_ + *β*^MCONC_0^_i_ + *β*^IBGROUP^_i_

Where P (X_i_) is the probability of the occurrence of symptomatic hypoglycemia given explanatory variables X_i_ where i correspond to the i^th^ individual, α is the effect*,* HBPR_0 is the indicator of average HbA1c (in %) for the i^th^ individual, PESO is the indicator of body weight (in kg) for the i^th^ individual, YEVDBT is the indicator of the time since diabetes diagnosis (in years) for the i^th^ individual, IBDOS_0 is the indicator of basal insulin daily dose (in IU) for the i^th^ individual, RENDBT_0 is the indicator of the presence of diabetic nephropathy for the i^th^ individual, ESTEAT_0 is the indicator of fatty liver disease (steatosis or steatohepatitis) for the i^th^ individual, MCONC_0 is the indicator of use of any concomitant oral antidiabetic medication(s) for the i^th^ individual and IBGROUP is the indicator of group (previous initiation of basal insulin) for the i^th^ individual.

Modeling started with the initial complete model (i.e., adjusting all covariates) and a backward selection method was applied for the selection of variables (final model).

Initial model (adjusting all variables) showed that the decrease in the body weight (p=0.011) and the increase in the basal insulin dose (p=0.005) are associated with an increase in the odds of developing symptomatic hypoglycemia.

| **Variable name** | **Variable label** | **OR** | **95% CI** | **p-value** |
| --- | --- | --- | --- | --- |
| HBPR | HbA1c (average) | 2.403 | 0.058 – 101.815 | 0.647 |
| PESO | Body weight (kg) | 0.970 | 0.947 – 0.993 | **0.011** |
| IBDOS_0 | Basal insulin dose (IU) | 1.024 | 1.007 – 1.041 | **0.005** |
| YEVDBT | Time since diabetes diagnosis (years) | 1.019 | 0.980 – 1.059 | 0.351 |
| RENDBT_0 | Diabetic nephropathy | 0.688 | 0.216 – 2.192 | 0.527 |
| ESTEAT_0 | Fatty liver disease | 0.155 | 0.020 – 1.173 | 0.071 |
| MCONC_0 | Concomitant use of any oral antidiabetic medication(s) | 1.504 | 0.539 – 4.198 | 0.436 |
| IBGROUP ^§^ | Group | 0.648 | 0.311 – 1.351 | 0.247 |

Final model also evidenced association between these variables and increase in the odds of symptomatic hypoglycemia.

Table. Multivariable analysis of symptomatic hypoglycemia factors (final model)

| **Variable name** | **Variable label** | **OR** | **95% CI** | **p-value** |
| --- | --- | --- | --- | --- |
| PESO | Body weight (kg) | 0.969 | 0.947 – 0.992 | **0.008** |
| IBDOS_0 | Basal insulin daily dose (IU) | 1.024 | 1.007 – 1.040 | **0.005** |
| ESTEAT_0 | Fatty liver disease | 0.140 | 0.019 – 1.051 | 0.056 |

*OR: odds ratio; CI: confidence interval; Goodness-of-fit: AIC: 253.393; -2 Log-likelihood 245.393; Hosmer-Lemeshow-X^2^ 11.052; p=0.199*

The final model showed that:

- The odds of developing symptomatic hypoglycemia for a person who has a 1 kg increase in the body weight over the odds of developing hypoglycemia for a person without weight increase is 0.969 (-3.1%), holding the rest of the variables at a fixed value.
- The odds of developing symptomatic hypoglycemia for a person who has a 1 IU increase in the daily dose of basal insulin dose over the odds of someone who did not have insulin dose increase is 1.024 (+2.4%), holding the rest of the variables at a fixed value.
- Note that a limitation of this multivariable analysis was the low quantity of symptomatic hypoglycemic events reported at Week 12.

**Primary analysis**

- The primary endpoint was performed at Week 12. The percentage of patients with at least 1 symptomatic hypoglycemia episode were provided.
- Four multivariable logistic regression models were used to analyze the impact of covariates (such as HbA1c, body weight, insulin dose, whether the patient has been recently initiated, diabetes years of evolution, renal or hepatic failure, concomitant antihyperglycemic medication) in the presence of hypoglycaemia.

**Secondary analysis**

Multivariable analyses were conducted with the purpose of:

- Identifying screening visit factors as predictive factors associated with treatment failure defined as failure to reach the individual target at Week 24.
- Identifying screening visit factors as predictive factors associated with treatment failure defined as failure to reach the general target <7.0% at Week 24.
- Factors associated with the failure to reach individual/general targets will be analyzed using a backward logistic regression. Factors such as baseline characteristics (demography [age, sex], history of diabetes [time since type 2 diabetes diagnosis], diabetes complications [Yes/No], physical examination [BMI], laboratory data [HbA1c within 1 month before study entry, FPG at study entry]...) will be considered and tested

**Fear of hypoglycemia survey**

**Encuesta de Miedo a la Hipoglucemia II**

| Encuesta de Nivel de Azúcar en Sangre Bajo en Adultos  (Universidad de Virginia)  I. Comportamiento: A continuación se presenta una lista de cosas que personas con diabetes en ocasiones hacen con el fin de evitar tener azúcar baja en la sangre y sus consecuencias. Circular el número a la derecha que mejor describa lo que ha realizado durante los últimos 6 meses en su rutina diaria para EVITAR niveles bajos de azúcar en sangre y sus consecuencias. (¡**Favor de no omitir ninguno!**).   \|  \| Nunca \| Rara vez \| En ocasiones \| Usualmente \| Casi siempre \| \| --- \| --- \| --- \| --- \| --- \| --- \| \| Para evitar niveles bajos de azúcar en sangre y como esto me afecta, yo…  1. Como snacks grandes  2. Trato de mantener mi azúcar en sangre por encima de 150.  3. Reduzco mi insulina cuando mi azúcar en sangre fue baja.  4. Mido mi azúcar en sangre seis o más veces al día.  5. Me aseguro que tenga alguien conmigo cuando salgo.  6. Limito mis salidas /viajes.  7. Limito las veces que manejo (coche, camioneta o bicicleta).  8. Evito visitar a amigos.  9. Permanezco en casa más de lo que quisiera.  10. Limito mi ejercicio/actividad física.  11. Me aseguro que hayan otras personas alrededor.  12. Evito tener sexo.  13. Mantengo mi azúcar en sangre más alta de lo usual en situaciones sociales.  14. Mantengo mi azúcar en sangre más alta de lo usual cuando hago tareas importantes.  15. Hago que las personas me revisen varias veces al día durante el día o la noche. \| 0  0  0  0  0  0  0  0  0  0  0  0  0  0  0 \| 1  1  1  1  1  1  1  1  1  1  1  1  1  1  1 \| 2  2  2  2  2  2  2  2  2  2  2  2  2  2  2 \| 3  3  3  3  3  3  3  3  3  3  3  3  3  3  3 \| 4  4  4  4  4  4  4  4  4  4  4  4  4  4  4 \| |
| --- | --- | --- | --- | --- | --- | --- | --- | --- | --- | --- | --- | --- |

| Encuesta de Nivel de Azúcar en Sangre Bajo en Adultos  (Universidad de Virginia)  II. Preocupación: A continuación se presenta una lista de preocupaciones que las personas con diabetes en ocasiones tienen acerca de sus valores bajos de azúcar en sangre. Favor de leer cada punto cuidadosamente (no omitir ninguno). Circular el número a la derecha que mejor describa que tan usualmente en los últimos 6 meses se ha PREOCUPADO acerca de cada punto debido a sus valores bajos de azúcar en sangre.   \|  \| Nunca \| Rara vez \| En ocasiones \| Usualmente \| Casi siempre \| \| --- \| --- \| --- \| --- \| --- \| --- \| \| Debido a que mis valores de azúcar en sangre podrían disminuir, me preocupo de:  16. No reconocer/ darme cuenta de que estuve teniendo valores bajos de azúcar en sangre.  17. No tener alimento, fruta o jugo disponible.  18. Desmayarme en público.  19. Avergonzarme a mí mismo o a mis amigos en una situación social.  20. Tener un episodio hipoglucémico mientras estoy solo.  21. Parecer que soy estúpido o que estoy borracho.  22. Perder el control.  23. Que no haya nadie alrededor para ayudarme durante un episodio hipoglucémico.  24. Tener un episodio hipoglucémico mientras manejo.  25. Realizar un error o tener un accidente.  26. Obtener una mala evaluación o ser criticado.  27. Dificultad en pensar claramente cuando soy responsable de otros.  28. Sentirme mareado o con la cabeza ligera.  29. Accidentalmente lastimarme a mí mismo o a otros.  30. Lesionar o dañar permanentemente a mi salud o cuerpo.  31. Que el azúcar en sangre baja interfiera con cosas importantes que estaba realizando.  32. Volverme hipoglucémico durante el sueño.  33. Enojarme emocionalmente y tener dificultades para lidiar con esto. \| 0  0  0  0  0  0  0  0  0  0  0  0  0  0  0  0  0  0 \| 1  1  1  1  1  1  1  1  1  1  1  1  1  1  1  1  1  1 \| 2  2  2  2  2  2  2  2  2  2  2  2  2  2  2  2  2  2 \| 3  3  3  3  3  3  3  3  3  3  3  3  3  3  3  3  3  3 \| 4  4  4  4  4  4  4  4  4  4  4  4  4  4  4  4  4  4 \| |
| --- | --- | --- | --- | --- | --- | --- | --- | --- | --- | --- | --- | --- |
